# Assembly of mammalian oxidative phosphorylation complexes I–V and supercomplexes

**DOI:** 10.1042/EBC20170098

**Published:** 2018-07-20

**Authors:** Alba Signes, Erika Fernandez-Vizarra

**Affiliations:** MRC-Mitochondrial Biology Unit, University of Cambridge, Hills Road, Cambridge CB2 0XY, U.K.

**Keywords:** atp synthase, electron transport chain, mitochondria, oxidative phosphorylation, respiratory chain complex assembly

## Abstract

The assembly of the five oxidative phosphorylation system (OXPHOS) complexes in the inner mitochondrial membrane is an intricate process. The human enzymes comprise core proteins, performing the catalytic activities, and a large number of ‘supernumerary’ subunits that play essential roles in assembly, regulation and stability. The correct addition of prosthetic groups as well as chaperoning and incorporation of the structural components require a large number of factors, many of which have been found mutated in cases of mitochondrial disease. Nowadays, the mechanisms of assembly for each of the individual complexes are almost completely understood and the knowledge about the assembly factors involved is constantly increasing. On the other hand, it is now well established that complexes I, III and IV interact with each other, forming the so-called respiratory supercomplexes or ‘respirasomes’, although the pathways that lead to their formation are still not completely clear. This review is a summary of our current knowledge concerning the assembly of complexes I–V and of the supercomplexes.

## Introduction

The oxidative phosphorylation system (OXPHOS) of the mitochondrial inner membrane is composed of five enzymes (complexes I–V; cI–V). In mammals, they are all multimeric and, except for cII, have subunits encoded both in the mitochondrial genome (mtDNA) and the nuclear genome (nDNA). The mtDNA-encoded subunits are hydrophobic and their translation happens close to the inner membrane to facilitate their translocation [1]. The nuclear-encoded structural subunits and many other factors necessary for the correct biogenesis of OXPHOS are expressed in the cytoplasm and imported inside the organelle [2].

Assembly of mitochondrial complexes II–V has been extensively studied in *Saccharomyces cerevisiae* [3–7], whereas research concerning cI has been carried out in *Yarrowia lipolytica* [8] and *Neurospora crassa* [9]. Many factors and mechanisms are conserved in mammals, and this has helped to identify genetic mutations associated with mitochondrial disease. However, it is now evident that there are specific factors in higher animals that are also involved in OXPHOS biogenesis and efforts are being made to understand their exact functions and implications in disease (see article by Ghezzi and Zeviani in this issue [201]). Moreover, studying assembly defects both in human cells and mouse disease models, has given highly valuable information about the assembly pathways and the proteins involved [10].

The OXPHOS complexes can interact with each other forming higher order structures, called supercomplexes or ‘respirasomes’ [11–13], whose functional role and assembly are still not completely understood [14–18].

## Assembly of complex I

Complex I (EC 1.6.5.3) or NADH:ubiquinone reductase (H^+^ translocating) with 45 subunits is the largest OXPHOS complex. It is an L-shaped enzyme with a hydrophilic arm protruding into the matrix, where electron transfer from NADH to coenzyme Q (CoQ) happens, and a proton translocating hydrophobic arm. The CoQ binding site is in the interface of both arms. Fourteen core subunits, conserved from bacteria to humans, perform the catalytic activities [19,20]. Seven core subunits in the hydrophilic arm contain the redox active centres: a non-covalently bound FMN and seven Fe–S clusters [21]. The other seven are all the cI subunits encoded in the mtDNA and are located in the hydrophobic arm, forming the proton channels [22]. The remaining 30 subunits are ‘supernumerary’ but important for assembly and stability [22–24].

**Figure 1:**
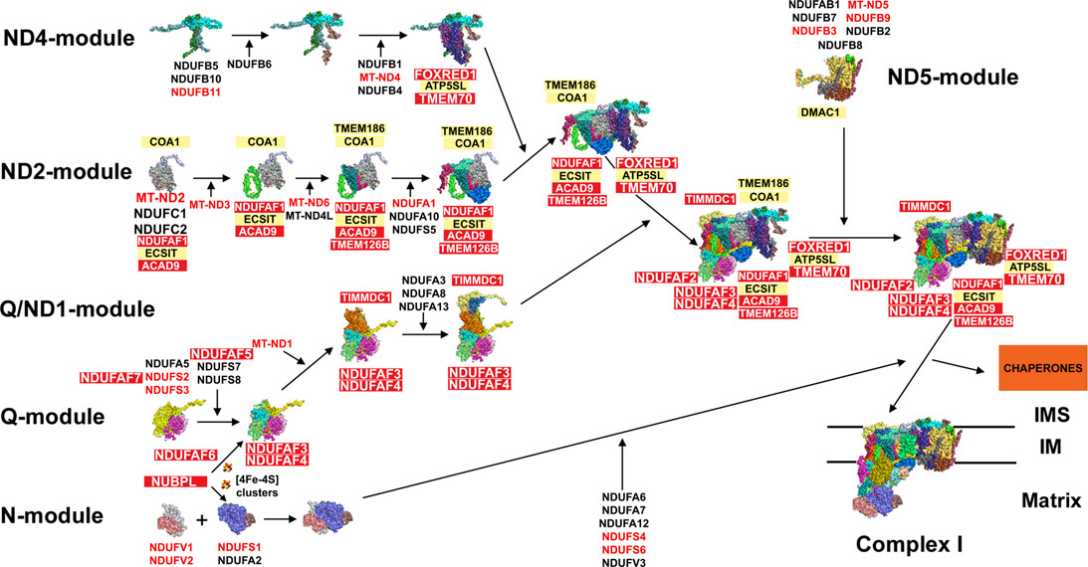
Complex I assembly model (see main text for details) based on the bovine cI cryo-EM structure with Protein Data Bank (PDB) ID: 5LC5 [23] and the models proposed in references [33,34,199] Red colour indicates proteins with described pathological mutations. Abbreviations: IM, inner membrane; IMS, intermembrane space.

Exhaustive research concerning human cI assembly has been carried out for 15 years [25–33]. However, several recent breakthroughs have granted a much deeper understanding about this process. Thus, we now know the complete mammalian cI structure [22,23] and how the subunits are organized in six modules (N, Q, ND1, ND2, ND4 and ND5) that, with the help of specific assembly factors, are brought together through five main subassemblies (Figure 1) [24,34].

The **N-module**, which is the tip of the hydrophilic arm and the last one to be incorporated [30,35], results from the assembly of NDUFV1, NDUFV2, NDUFS1 and NDUFA2 [34], to which NDUFA6, NDUFA7, NDUFA12, NDUFS4, NDUFS6 and NDUFV3 must be further associated with to complete the module [24].

The **Q-module** is built through the association of NDUFA5, NDUFS2 and NDUFS3 plus NDUFS7 and NDUFS8. The chaperones NDUFAF3/C3ORF60 and NDUFAF4/C6ORF66 [36,37] remain bound to this module until the final assembly steps [34]. NDUFAF6/C8ORF38 [38] also seems to participate in the assembly of the Q-module [24,39]. NDUFAF3, 4 and 6, are necessary to maintain normal MT-ND1 synthesis [40,41]. NDUFAF5 adds a hydroxyl group to Arg^73^ of NDUFS7 [42] and NDUFAF7 dimethylates NDUFS2 in Arg^85^ [43], an essential modification for cI assembly [44]. NUBPL/IND1 delivers [4Fe–4S] clusters specifically to the N- and Q-module subunits [45,46].

The **ND1-module** builds around the Q-module with the help of TIMMDC1/C3ORF1 [47,48], which remains bound to the Q/ND1 subassembly until the last maturation steps. MT-ND1 joins first and then NDUFA3, NDUFA8 and NDUFA13 are added [34].

The **ND2-module** is formed by an initial intermediate that contains MT-ND2, NDUFC1 and NDUFC2 bound to NDUFAF1/CIA30 [49,50], ECSIT [51] and ACAD9 [52,53]. Then, MT-ND3 is added together with TMEM126B [54], forming a larger intermediate to which subunits MT-ND6 and MT-ND4L bind. The latest assembly stages involve the incorporation of subunits NDUFA1, NDUFA10 and NDUFS5 [24,34]. The stable association of the assembly factors NDUFAF1 + ECSIT + ACAD9 + TMEM126 was denominated Mitochondrial Complex I Assembly (MCIA) complex [48,54]. Two other chaperones were found interacting with this module: TMEM186 and COA1 [34], the latter being a well-known cIV assembly factor [55,56].

The main **ND4-module** intermediate binds NDUFB1, NDUFB4, NDUFB5, NDUFB6, NDUFB10, NDUFB11 and MT-ND4 together with FOXRED1 [46,57–59], ATP5SL [24,47] and also TMEM70, described as a cV assembly factor [34,60,61].

The **ND5-module** corresponds to the distal part of the membrane arm and it is composed of MT-ND5, NDUFB2, NDUFB3, NDUFB7, NDUFB8, NDUFB9 and NDUFAB1 [24,34]. DMAC1/TMEM261 is implicated in its stabilization and/or assembly [24].

The ND2- and the ND4-modules get together first, with still all the chaperones bound to them. Later on, the Q/ND1 and the ND5-modules join the nascent complex. This intermediate only lacking the N-module is stabilized by NDUFAF2/NDUFA12L/B17.2L [24,35,62]. In the last step, the pre-assembled N-module becomes attached and the chaperones released [34].

## Assembly of complex II

Complex II (EC 1.3.5.1) or succinate dehydrogenase (quinone) is shared between the TCA cycle and the ETC and has no proton pumping activity. It is composed of four nDNA-encoded subunits. The two hydrophilic catalytic subunits are SDHA/SDH1 and SDHB/SDH2. Hydrophobic subunits SDHC/SDH3 and SDHD/SDH4 constitute the cII membrane anchor, containing a haem *b* group and two CoQ binding sites [63–65]. The two electrons from succinate oxidation are transferred to a FAD covalently bound to SDHA, then to the three different Fe–S clusters in SDHB and finally to CoQ [65,66].

**Figure 2:**
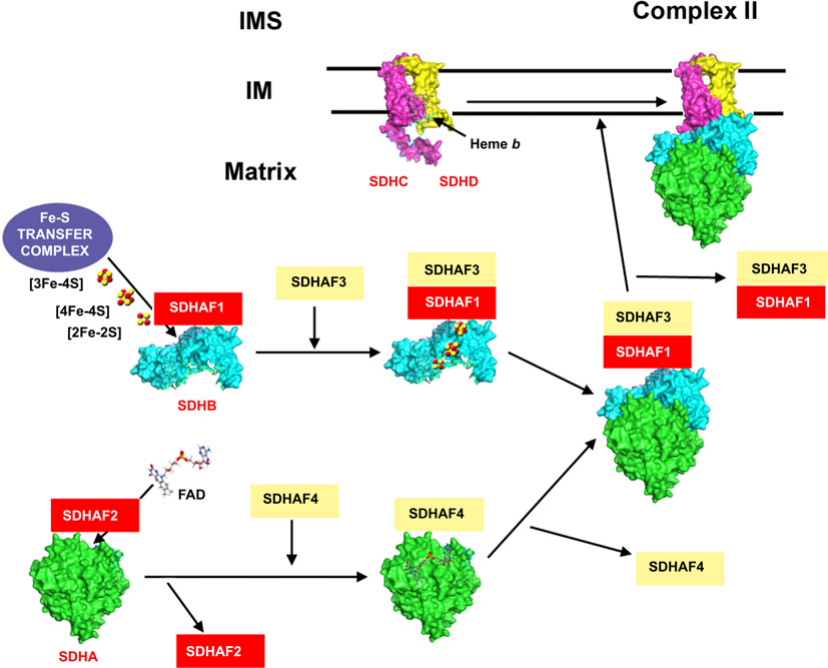
Complex II assembly model (see main text for details) based on the porcine cII crystal structure with PDB ID: 1ZOY [65] and the model proposed in reference [7] Red colour indicates proteins with described pathological mutations. Abbreviations: IM, inner membrane; IMS, intermembrane space.

Complex II assembly (Figure 2) happens through the independent maturation of SDHA, SDHB and SDHC + SDHD mediated by subunit-specific chaperones [7].

**SDHA** is flavinylated before assembly into cII, and SDHAF2/Sdh5 mediates this step [67,68]. Following FAD incorporation, SDHA binds to SDHAF4/Sdh8, which keeps the subunit stable and competent for assembly with SDHB, while protecting it from auto-oxidation [69].

**SDHB** also incorporates its Fe–S clusters before joining the rest of the subunits. Fe–S clusters are synthesized in the mitochondrial matrix [70,71] and then transferred to the apoprotein. This step is mediated by SDHAF1, necessary also for SDHB stability [72–74]. SDHAF3/ACN9/LYRM10 is another protein involved in SDHB stability and oxidative damage protection after insertion of the Fe–S clusters [7,75,76].

When both SDHA and SDHB acquire their respective prosthetic groups they join together, liberating SDHAF4 but keeping the binding with SDHAF1 and SDHAF3 [7,75].

**SDHC** and **SDHD** are assembled together in the inner membrane by a yet unknown mechanism. The haem *b* group, co-ordinated in the interface of both subunits, does not play any catalytic role but is required for their stability [77,78]. Another factor that influences the dimerization of SDHC and SDHD, as well as their stability, is the presence of both hydrophilic subunits [68,75].

## Assembly of complex III

Complex III (EC 1.10.2.2) or quinol-cytochrome *c* reductase performs electron transfer coupled to proton pumping using the ‘Q-cycle’ mechanism [79,80]. Structurally, it is a tightly bound symmetrical dimer (cIII_2_), being each ‘monomer’ composed of three catalytic core (MT-CYB, CYC1 and UQCRFS1) and seven supernumerary subunits [81,82]. The 78-amino acid mitochondrial targeting sequence (MTS) cleaved off from UQCRFS1 was considered an extra subunit [81,83], but it needs to be cleared out to maintain cIII_2_ structural and functional fitness [84,85]. MT-CYB contains two *b*-type haems with different redox potential as well as two CoQ binding sites. There is one [2Fe–2S] cluster inserted in the C-terminal end of UQCRFS1, and CYC1 binds a haem *c1* group that transfers the electrons to the mobile electron carrier cytochrome *c*. The supernumerary subunits are not involved in the catalysis, but are important for correct assembly and/or stability of the enzyme [86,87].

**Figure 3:**
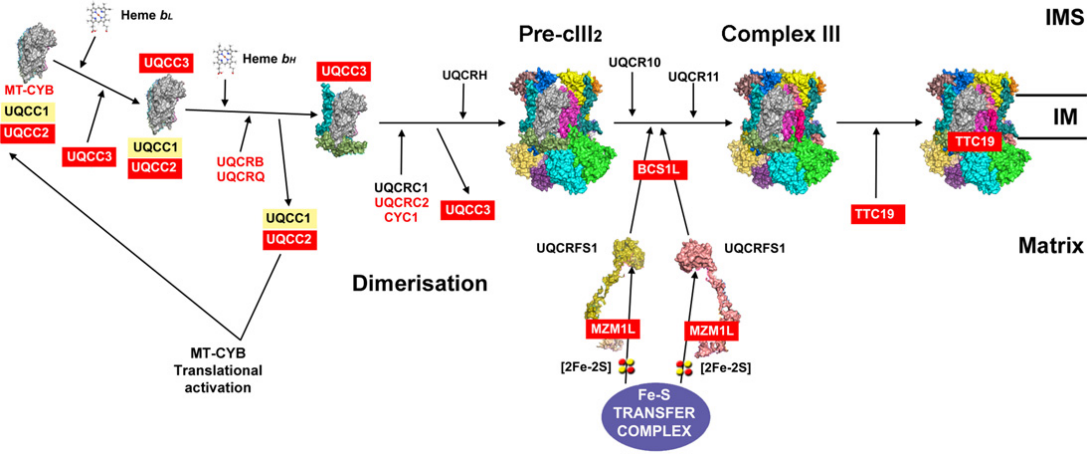
Complex III assembly model (see main text for details) based on the bovine cIII_2_ crystal structure with PDB ID: 1BGY [81] and the models proposed in references [85,103] Red colour indicates proteins with described pathological mutations. Abbreviations: IM, inner membrane; IMS, intermembrane space.

Yeast cIII assembly starts with the synthesis of **cytochrome *b*** (MT-CYB in human nomenclature) by mitochondrial ribosomes and its insertion into the inner membrane, mediated by Cbp3/UQCC1 and Cbp6/UQCC2 that remain bound to MT-CYB once it is completely synthesized. Cbp4/UQCC3 joins after the first haem-*b* (*b*_L_) but before the second one (*b*_H_) is incorporated [88–90]. Once the first structural subunits (UQCRB and UQCRQ) are incorporated, UQCC1-UQCC2 detach and go back to act as translational activators [88,89]. These first steps in cIII assembly (Figure 3) are supposedly conserved, because the three factors are present in humans and mutations in *UQCC2* produce deficient MT-CYB synthesis [91,92].

Maturation of cIII occurs, both in yeast and humans, with the addition of the **Rieske Fe–S protein** (Rip1/UQCRFS1) and of the smallest subunit (Qcr10/UQCR11) to an already dimeric pre-complex III (pre-cIII_2_) [93–95]. After import into mitochondria, UQCRFS1 is bound and stabilized in the matrix by MZM1L/LYRM7 [96–98] that also mediates binding to the Fe–S cluster transfer complex [99]. Incorporation of UQCRFS1 to pre-cIII_2_ is mediated by Bcs1/BCS1L [93,94,100,101]. In human and mouse mitochondria, TTC19 [102] binds fully assembled cIII_2_ and favours the elimination of UQCRFS1 N-terminal fragments to maintain normal activity levels [84].

The intermediate steps of cIII_2_ assembly are not known in humans. However, being that the initial and the final stages are the same and the assembly factors involved are orthologous proteins, it is assumed that they will share very many similarities [103]. The order of incorporation in *S. cerevisiae* was determined by creating yeast strains deleting one structural subunit at a time and studying the stability of the remaining cIII components [104–107]. Up to now, there are no described assembly factors involved in the incorporation or stabilization of cIII_2_ intermediate subunits and transitional subcomplexes.

## Assembly of complex IV

Complex IV (EC 1.9.31) or cytochrome *c* oxidase (COX) catalyses the oxidation of cytochrome *c* and the reduction of oxygen to water, coupled to proton translocation [108]. Mammalian cIV contains 13 or 14 subunits [109–111]. MT-CO1 is the largest catalytic subunit containing a haem *a* group and a binuclear haem *a_3_*-Cu_B_ centre. MT-CO2 is the second core subunit and holds the Cu_A_ centre. MT-CO3, the third core subunit, plays no direct catalytic role [108]. The rest of subunits (supernumerary) are thought to be important for the stabilization of the catalytic core and regulation of its activity [112–117]. Complex IV is the only OXPHOS complex containing tissue-specific and developmentally regulated isoforms [118,119], reflecting the importance of an exquisite regulation of COX activity.

**Figure 4:**
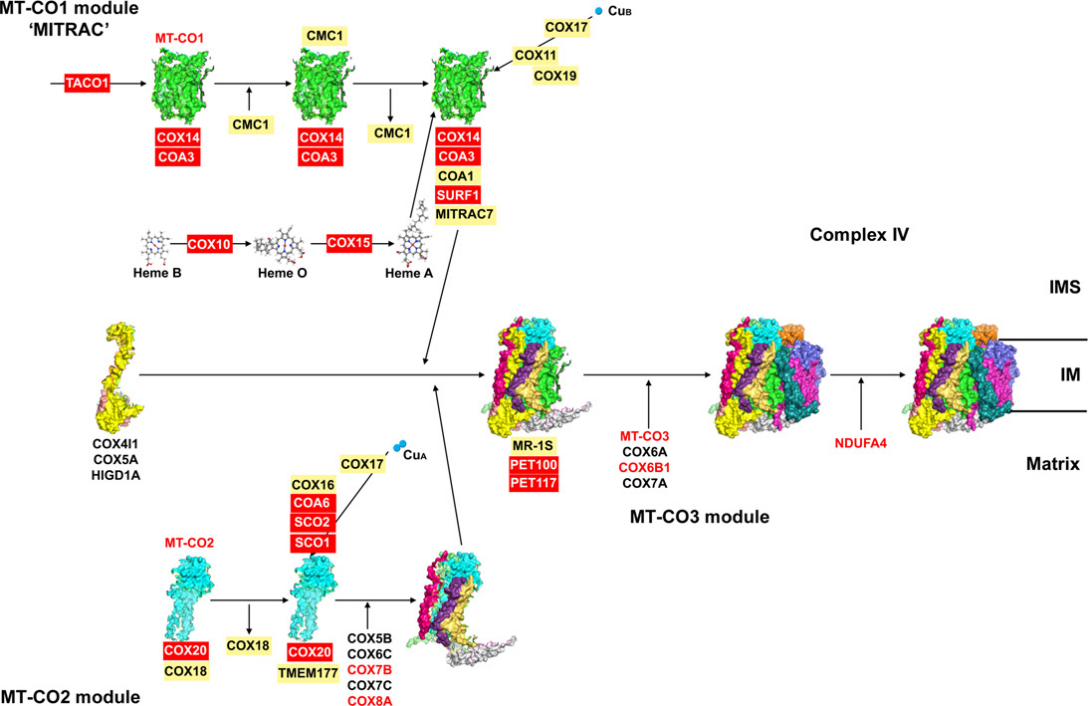
Complex IV assembly model (see main text for details) based on the bovine cIV crystal structure with PDB ID: 2OCC [109] and the model proposed in reference [123] Red colour indicates proteins with described pathological mutations. Abbreviations: IM, inner membrane; IMS, intermembrane space.

The first model of subunit incorporation for human COX [120], basically still stands with minor modifications [115,121–124]. According to this model, **MT-CO1** is the ‘seed’ around which the rest of the subunits build up, starting with COX4I1 and COX5A. The stable subassemblies created during this process were named S1–S4, S4 being the fully assembled holoenzyme [10,120]. Proteomics analyses of a MT-CO3-deficient cell line, with a very prominent subcomplex accumulation, completed the view about subunit incorporation (Figure 4), which happens in groups or ‘modules’, defined by each one of the core subunits [123], as it does in yeast [125].

The **initial COX subassembly** appears to be the association of COX4I1 + COX5A [123]. This early subcomplex also contains HIGD1A [123], one of the human homologues of yeast Rcf1 [126–129].

The **MT-CO1 module** contains the many chaperones and assembly factors involved in its maturation and stabilization, and it is also known as ‘MITRAC’ for mitochondrial translation regulation assembly intermediate of cytochrome *c* oxidase [56,130]. COX14/C12ORF62 [55,131] and COA3/CCDC56/MITRAC12 [56,132] bind nascent MT-CO1 and are implicated in assembly regulation either by translational [133] or post-translational mechanisms [134]. In human mitochondria, MT-CO1 expression is especially sensitive to defects in the mitochondrial RNA-binding protein LRPPRC [135–137] and requires the specific translational activator TACO1 [138,139]. Later on, CMC1 binds MT-CO1 + COA3 + COX14 before or during addition of the prosthetic groups [134]. Haem A biosynthesis is carried out by COX10 [140,141] and COX15 [142]. The exact molecular function of SURF1 [143,144] remains unclear, but its involvement in haem A delivery has been proposed [124]. A role for PET117 in this same process has been suggested due to its interaction with COX15 [145]. Cu_B_ assembly requires the metallochaperone COX11 [146,147], with COX17 donating the coppers [148,149], and COX19 maintaining COX11 in the right redox state [150]. CMC1 is released prior to the addition of COA1/C7ORF44/MITRAC15 [55,56,151] and SURF1. MITRAC7/SMIM20 is another factor described to stabilize MT-CO1 in early assembly stages [130].

The intermediate step in COX assembly is the joining of COX4I1 + COX5A, MT-CO1 and the **MT-CO2 module** (MT-CO2 + COX5B + COX6C + COX7C + COX8A and, most probably COX7B), equalling the ‘S3’ intermediary [120] minus MT-CO3 [123]. MT-CO2 requires COX18 for membrane translocation [152] and COX20/FAM36A and TMEM177 for stabilization [153–155]. Copper-binding proteins COX17, SCO1 and SCO2 [156–158] together with COA6 [159–161] and COX16 [162–164], are involved in the assembly of the Cu_A_ centre. MR-1S is a vertebrate-specific COX chaperone that interacts with the highly conserved factors PET100 [165–167] and PET117 [168,169] during assembly of the MT-CO2 module [123].

The incorporation of the **MT-CO3 module** (MT-CO3 + COX6A1 + COX6B1 + COX7A2) completes the assembly of the 13 canonical COX subunits [109,123]. No specific assembly factors for this module are currently known.

The **last subunit** to be incorporated is NDUFA4, initially thought to be part of complex I [170], but later assigned to complex IV [110,117].

More proteins that those described here are required for cIV assembly [124] but their exact molecular role is still not understood.

## Assembly of complex V

Complex V (EC 3.6.14), H^+^-transporting two-sector ATPase or F_o_F_1_-ATPase, is the enzyme that synthesizes ATP using the proton motive force generated by cI, III and IV. It is composed of two topological and functional distinct domains: membrane-extrinsic and matrix-facing F_1_ plus membrane-intrinsic F_o_, with a central axis and a peripheral stalk connecting them [171]. Subunits a (MT-ATP6) and A6L (MT-ATP8) of the F_o_ domain are encoded in the mtDNA, whereas all the rest of cV components are nDNA encoded [172]. Protons coming back to the matrix through F_o_ produce a rotational movement providing the energy for ADP+Pi condensation in the F_1_ domain [171,173].

**Figure 5:**
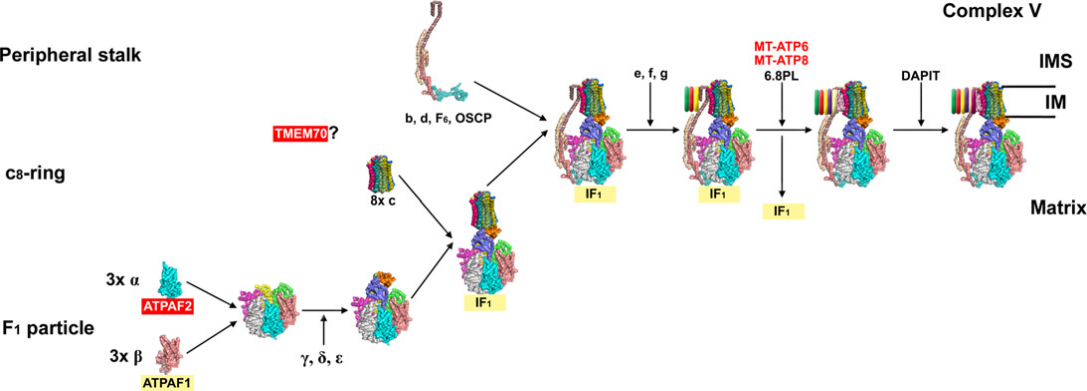
Complex V assembly model (see main text for details) based on the bovine cV cryo-EM structure with PDB ID: 5ARA [200] and the model proposed in references [172,180] Red colour indicates proteins with described pathological mutations. Abbreviations: IM, inner membrane; IMS, intermembrane space.

Assembly of cV has been studied using subunit incorporation dynamics [174], analysis of mtDNA-deficient cell lines [175,176] and more recently by creating knockout cell lines for specific cV subunits [177–180]. As depicted in Figure 5, this complex is also put together by assembling three pre-formed modules corresponding to: F_1_ particle, c_8_-ring (a ring composed by eight copies of the c-subunit) and peripheral stalk [172].

The **F_1_ subcomplex**, composed of three copies of the α subunit/ATP5A1, three β subunits/ATP5B together with the central stalk subunits γ/ATP5C1, δ/ATP5D and ε/ATP5E, is assembled with the assistance of chaperones ATPAF1/ATP11 and ATPAF2/ATP12, which bind ATP5B and ATP5A1, respectively [181–185]. The **c_8_-ring**, encoded by *ATPG1, ATPG2* and *ATPG3*, is assembled in the membrane by still unknown mechanisms [172]. A subcomplex containing subunits of the **peripheral stalk** is also pre-formed [177,180].

After the c_8_-ring and the F_1_ subcomplex come together, the peripheral stalk is incorporated in two steps: first subunits b/ATP5F1, d/ATPH, F_6_/ATP5J and OSCP/ATP5O and then e/ATP5I, g/ATP5L and f/ATPJ2 [172,180]. The peripheral stalk can also join the F_1_ subcomplex in absence of the c_8_-ring [179,180]. During these initial steps, the inhibitor protein IF_1_ is bound to the intermediates, being liberated with the insertion of the two mtDNA-encoded subunits [178–180]. In the cases in which a/MT-ATP6 and A6L/MT-ATP8 are missing, the previous assembly intermediate is readily accumulated [174,176,179]. The interaction of the last subunits is stabilized by 6.8L/MLQ/C14ORF2 and the peripheral subunit DAPIT/USMG5 is incorporated to finish cV assembly [180].

One of the few proteins known to be involved in cV biogenesis is TMEM70 and although its exact function is still not known, mutations in the gene encoding this factor have recurrently been associated with ATP synthase deficiency [60,186].

## Assembly of respiratory supercomplexes

The OXPHOS complexes interact with each other forming higher order structures, which have been called supercomplexes. Complexes IV and V can form dimers and oligomers [11,187,188]. In addition, defined associations of complexes I, III and IV are reproducibly found when mitochondrial membrane extracts are solubilized with digitonin and separated through Blue Native Gel Electrophoresis [11,12]. Thus, according to their molecular size and subunit composition, the main supercomplexes have been assigned the following stoichiometries: III_2_IV_1_, I_1_III_2_, I_1_III_2_IV_1_, and I_2_III_2_IV_1–2_. Supercomplex I_1_III_2_IV_1_ is the ‘respirasome’ and supercomplex I_2_III_2_IV_2_ has been named as ‘respiratory megacomplex’ [189]. High-resolution Cryo-EM structures of the respirasome of several mammalian species, including human, have been recently resolved [189–193]. The association of the individual complexes into these structurally defined supercomplexes is now very well established but their specific functional role still needs to be clarified [14–18].

Two alternative views exist to explain respirasome assembly. The first possibility is that the individual complexes are completely assembled before they join together in the supercomplexes [12,34]. This mode of action would permit the dynamic association-dissociation of the complexes to adapt to varying energy demands, if the role of the supercomplexes were to increase the efficiency of electron transfer, as proposed by the ‘plasticity model’ [12,194]. However, there are also evidences pointing to the co-assembly of subunits from the different complexes before completion of the single enzymes. Accordingly, maturation of cI would not happen unless cIII_2_ and cIV are bound to a ‘pre-cI’ scaffold [195]. Also, incomplete complexes have been found assembled together in cultured cells and tissues from patients carrying mutations in different structural subunits and assembly factors implicated in the last steps of cI and cIII assembly [10]. The fact that COA1, a well-characterized cIV chaperone, is bound to cI assembly intermediates [34] could also reflect co-assembly of at least cI and cIV, although the authors of this report did not provide evidence as to whether MT-CO1 is also bound to the same subcomplexes.

COX7A2L/COX7R/SCAFI is an orthologue of the cIV structural subunit COX7A that was first described as a supercomplex assembly factor because of being necessary for the incorporation of cIV into supercomplex structures [194]. However, more recent evidences have demonstrated a role for this protein for the formation of III_2_IV_1_ but not for the incorporation of cIV into the respirasomes [188,196,197]. The dynamic interchange between the three types of COX7A proteins, COX7A2L (SCAFI), COX7A1 (muscle-type structural subunit) and COX7A2 (liver-type structural subunit) could potentially determine whether cIV stays as a monomer, oligomerizes or forms the III_2_IV_1_ supercomplex, as well as the mode of binding to cI [13,198].

## Final remarks

Assembly of the OXPHOS system is an intricate process that we still do not completely understand, despite the great efforts of many research teams and the spectacular advances described here. It is important to continue studying the processes governing the assembly of each of the complexes and of the supercomplexes, as well as the exact molecular role of the proteins involved in its basic assembly and fine regulation. This will help us understand the mechanisms regulating this central part of metabolism in health and disease. For a detailed explanation of the pathologies associated with mutations in the described assembly factors, see the accompanying article in this issue: ‘Human diseases associated with defects in assembly of OXPHOS complexes’ Ghezzi and Zeviani [201].

## Summary

- Assembly of the OXPHOS complexes requires a significant amount of ancillary proteins.
- Many assembly factors are conserved from yeast to humans, but some are specific for higher animals.
- Complex I is the largest OXPHOS enzyme and its assembly occurs through modules, each of which requires specific assembly factors.
- Despite being the smallest OXPHOS component, complex II assembly is assisted by, at least, four different chaperones.
- Up to now, only the first and last steps of complex III assembly are well understood.
- Complex IV assembly is highly regulated, with more than 30 known assembly factors, involved mainly in the maturation of the catalytic core.
- The order of incorporation of the 17 subunits of complex V is well known, but only 3 assembly factors have been identified so far.
- The OXPHOS complexes interact with each other in the supercomplexes or ‘respirasomes’, although the way they assemble together is still not known.

## Abbreviations

CoQ: coenzyme Q
COX: cytochrome *c* oxidase
MITRAC: mitochondrial translation regulation assembly intermediate of cytochrome *c* oxidase
MTS: mitochondrial targeting sequence
OXPHOS: oxidative phosphorylation system

## References

[1] Mai N., Chrzanowska-Lightowlers Z.M. and Lightowlers R.N. (2017) The process of mammalian mitochondrial protein synthesis. Cell Tissue Res. 367, 5–20 10.1007/s00441-016-2456-0 27411691 PMC5203842

[2] Wasilewski M., Chojnacka K. and Chacinska A. (2017) Protein trafficking at the crossroads to mitochondria. Biochim. Biophys. Acta 1864, 125–137 10.1016/j.bbamcr.2016.10.019 27810356

[3] Tzagoloff A. and Dieckmann C.L. (1990) PET genes of *Saccharomyces cerevisiae*. Microbiol. Rev. 54, 211–225 2215420 10.1128/mr.54.3.211-225.1990 PMC372773

[4] Barrientos A. (2003) Yeast models of human mitochondrial diseases. IUBMB Life 55, 83–95 10.1002/tbmb.718540876 12749690

[5] Fontanesi F., Soto I.C., Horn D. and Barrientos A. (2006) Assembly of mitochondrial cytochrome c-oxidase, a complicated and highly regulated cellular process. Am. J. Physiol. Cell Physiol. 291, C1129–C47 10.1152/ajpcell.00233.2006 16760263

[6] Smith P.M., Fox J.L. and Winge D.R. (2012) Biogenesis of the cytochrome bc(1) complex and role of assembly factors. Biochim. Biophys. Acta 1817, 276–286 10.1016/j.bbabio.2011.11.009 22138626 PMC3366459

[7] Van Vranken J.G., Na U., Winge D.R. and Rutter J. (2015) Protein-mediated assembly of succinate dehydrogenase and its cofactors. Crit. Rev. Biochem. Mol. Biol. 50, 168–180 10.3109/10409238.2014.990556 25488574 PMC4653115

[8] Kerscher S., Drose S., Zwicker K., Zickermann V. and Brandt U. (2002) *Yarrowia lipolytica*, a yeast genetic system to study mitochondrial complex I. Biochim. Biophys. Acta 1555, 83–91 10.1016/S0005-2728(02)00259-1 12206896

[9] Schulte U. (2001) Biogenesis of respiratory complex I. J. Bioenerg. Biomembr. 33, 205–212 10.1023/A:1010730919074 11695830

[10] Fernandez-Vizarra E., Tiranti V. and Zeviani M. (2009) Assembly of the oxidative phosphorylation system in humans: what we have learned by studying its defects. Biochim. Biophys. Acta 1793, 200–211 10.1016/j.bbamcr.2008.05.028 18620006

[11] Schagger H. (2002) Respiratory chain supercomplexes of mitochondria and bacteria. Biochim. Biophys. Acta 1555, 154–159 10.1016/S0005-2728(02)00271-2 12206908

[12] Acin-Perez R., Fernandez-Silva P., Peleato M.L., Perez-Martos A. and Enriquez J.A. (2008) Respiratory active mitochondrial supercomplexes. Mol. Cell 32, 529–539 10.1016/j.molcel.2008.10.021 19026783

[13] Letts J.A. and Sazanov L.A. (2017) Clarifying the supercomplex: the higher-order organization of the mitochondrial electron transport chain. Nat. Struct. Mol. Biol. 24, 800–808 10.1038/nsmb.3460 28981073

[14] Barrientos A. and Ugalde C. (2013) I function, therefore i am: overcoming skepticism about mitochondrial supercomplexes. Cell Metab. 18, 147–149 10.1016/j.cmet.2013.07.010 23931749 PMC3912836

[15] Acin-Perez R. and Enriquez J.A. (2014) The function of the respiratory supercomplexes: the plasticity model. Biochim. Biophys. Acta 1837, 444–450 10.1016/j.bbabio.2013.12.009 24368156

[16] Moreno-Loshuertos R. and Enriquez J.A. (2016) Respiratory supercomplexes and the functional segmentation of the CoQ pool. Free Radic. Biol. Med. 100, 5–13 10.1016/j.freeradbiomed.2016.04.018 27105951

[17] Lobo-Jarne T. and Ugalde C. (2018) Respiratory chain supercomplexes: structures, function and biogenesis. Semin. Cell Dev. Biol., 76, 179–190, 10.1016/j.semcdb.2017.07.021 28743641 PMC5780262

[18] Milenkovic D., Blaza J.N., Larsson N.G. and Hirst J. (2017) The enigma of the respiratory chain supercomplex. Cell Metab. 25, 765–776 10.1016/j.cmet.2017.03.009 28380371

[19] Efremov R.G., Baradaran R. and Sazanov L.A. (2010) The architecture of respiratory complex I. Nature 465, 441–445 10.1038/nature09066 20505720

[20] Baradaran R., Berrisford J.M., Minhas G.S. and Sazanov L.A. (2013) Crystal structure of the entire respiratory complex I. Nature 494, 443–448 10.1038/nature11871 23417064 PMC3672946

[21] Hirst J. and Roessler M.M. (2016) Energy conversion, redox catalysis and generation of reactive oxygen species by respiratory complex I. Biochim. Biophys. Acta 1857, 872–883 10.1016/j.bbabio.2015.12.009 26721206 PMC4893023

[22] Vinothkumar K.R., Zhu J. and Hirst J. (2014) Architecture of mammalian respiratory complex I. Nature 515, 80–84 10.1038/nature13686 25209663 PMC4224586

[23] Zhu J., Vinothkumar K.R. and Hirst J. (2016) Structure of mammalian respiratory complex I. Nature 536, 354–358 10.1038/nature19095 27509854 PMC5027920

[24] Stroud D.A., Surgenor E.E., Formosa L.E., Reljic B., Frazier A.E., Dibley M.G. (2016) Accessory subunits are integral for assembly and function of human mitochondrial complex I. Nature 538, 123–126 10.1038/nature19754 27626371

[25] Antonicka H., Ogilvie I., Taivassalo T., Anitori R.P., Haller R.G., Vissing J. (2003) Identification and characterization of a common set of complex I assembly intermediates in mitochondria from patients with complex I deficiency. J. Biol. Chem. 278, 43081–43088 10.1074/jbc.M304998200 12941961

[26] Ugalde C., Janssen R.J., van den Heuvel L.P., Smeitink J.A. and Nijtmans L.G. (2004) Differences in assembly or stability of complex I and other mitochondrial OXPHOS complexes in inherited complex I deficiency. Hum. Mol. Genet. 13, 659–667 10.1093/hmg/ddh071 14749350

[27] Ugalde C., Vogel R., Huijbens R., van den Heuvel B., Smeitink J. and Nijtmans L. (2004) Human mitochondrial complex I assembles through the combination of evolutionary conserved modules: a framework to interpret complex I deficiencies. Hum. Mol. Genet. 13, 2461–2472 10.1093/hmg/ddh262 15317750

[28] Vogel R.O., Dieteren C.E., van den Heuvel L.P., Willems P.H., Smeitink J.A., Koopman W.J. (2007) Identification of mitochondrial complex I assembly intermediates by tracing tagged NDUFS3 demonstrates the entry point of mitochondrial subunits. J. Biol. Chem. 282, 7582–7590 10.1074/jbc.M609410200 17209039

[29] Vogel R.O., Smeitink J.A. and Nijtmans L.G. (2007) Human mitochondrial complex I assembly: A dynamic and versatile process. Biochim. Biophys. Acta 1767, 1215–1227 10.1016/j.bbabio.2007.07.008 17854760

[30] Lazarou M., McKenzie M., Ohtake A., Thorburn D.R. and Ryan M.T. (2007) Analysis of the assembly profiles for mitochondrial- and nuclear-DNA-encoded subunits into complex I. Mol. Cell Biol. 27, 4228–4237 10.1128/MCB.00074-07 17438127 PMC1900046

[31] Lazarou M., Thorburn D.R., Ryan M.T. and McKenzie M. (2009) Assembly of mitochondrial complex I and defects in disease. Biochim. Biophys. Acta 1793, 78–88 10.1016/j.bbamcr.2008.04.015 18501715

[32] Mimaki M., Wang X., McKenzie M., Thorburn D.R. and Ryan M.T. (2012) Understanding mitochondrial complex I assembly in health and disease. Biochim. Biophys. Acta 1817, 851–862 10.1016/j.bbabio.2011.08.010 21924235

[33] Sanchez-Caballero L., Guerrero-Castillo S. and Nijtmans L. (2016) Unraveling the complexity of mitochondrial complex I assembly: a dynamic process. Biochim. Biophys. Acta 1857, 980–990 10.1016/j.bbabio.2016.03.031 27040506

[34] Guerrero-Castillo S., Baertling F., Kownatzki D., Wessels H.J., Arnold S., Brandt U. (2017) The assembly pathway of mitochondrial respiratory chain complex I. Cell Metab. 25, 128–139 10.1016/j.cmet.2016.09.002 27720676

[35] Vogel R.O., van den Brand M.A., Rodenburg R.J., van den Heuvel L.P., Tsuneoka M., Smeitink J.A. (2007) Investigation of the complex I assembly chaperones B17.2L and NDUFAF1 in a cohort of CI deficient patients. Mol. Genet. Metab. 91, 176–182 10.1016/j.ymgme.2007.02.007 17383918

[36] Saada A., Edvardson S., Rapoport M., Shaag A., Amry K., Miller C. (2008) C6ORF66 is an assembly factor of mitochondrial complex I. Am. J. Hum. Genet. 82, 32–38 10.1016/j.ajhg.2007.08.003 18179882 PMC2253982

[37] Saada A., Vogel R.O., Hoefs S.J., van den Brand M.A., Wessels H.J., Willems P.H. (2009) Mutations in NDUFAF3 (C3ORF60), encoding an NDUFAF4 (C6ORF66)-interacting complex I assembly protein, cause fatal neonatal mitochondrial disease. Am. J. Hum. Genet. 84, 718–727 10.1016/j.ajhg.2009.04.020 19463981 PMC2694978

[38] Pagliarini D.J., Calvo S.E., Chang B., Sheth S.A., Vafai S.B., Ong S.E. (2008) A mitochondrial protein compendium elucidates complex I disease biology. Cell 134, 112–123 10.1016/j.cell.2008.06.016 18614015 PMC2778844

[39] Bianciardi L., Imperatore V., Fernandez-Vizarra E., Lopomo A., Falabella M., Furini S. (2016) Exome sequencing coupled with mRNA analysis identifies NDUFAF6 as a Leigh gene. Mol. Genet. Metab., 10.1016/j.ymgme.2016.09.001 27623250

[40] McKenzie M., Tucker E.J., Compton A.G., Lazarou M., George C., Thorburn D.R. (2011) Mutations in the gene encoding C8orf38 block complex i assembly by inhibiting production of the mitochondria-encoded subunit ND1. J. Mol. Biol. 414, 413–426 10.1016/j.jmb.2011.10.012 22019594

[41] Zurita Rendon O. and Shoubridge E.A. (2012) Early complex I assembly defects result in rapid turnover of the ND1 subunit. Hum. Mol. Genet. 21, 3815–3824 10.1093/hmg/dds209 22653752 PMC3412381

[42] Rhein V.F., Carroll J., Ding S., Fearnley I.M. and Walker J.E. (2016) NDUFAF5 hydroxylates NDUFS7 at an early stage in the assembly of human complex I. J. Biol. Chem. 291, 14851–14860 10.1074/jbc.M116.734970 27226634 PMC4938201

[43] Rhein V.F., Carroll J., Ding S., Fearnley I.M. and Walker J.E. (2013) NDUFAF7 methylates arginine 85 in the NDUFS2 subunit of human complex I. J. Biol. Chem. 288, 33016–33026 10.1074/jbc.M113.518803 24089531 PMC3829151

[44] Zurita Rendon O., Silva Neiva L., Sasarman F. and Shoubridge E.A. (2014) The arginine methyltransferase NDUFAF7 is essential for complex I assembly and early vertebrate embryogenesis. Hum. Mol. Genet. 23, 5159–5170 10.1093/hmg/ddu239 24838397 PMC4159157

[45] Sheftel A.D., Stehling O., Pierik A.J., Netz D.J., Kerscher S., Elsasser H.P. (2009) Human ind1, an iron-sulfur cluster assembly factor for respiratory complex I. Mol. Cell. Biol. 29, 6059–6073 10.1128/MCB.00817-09 19752196 PMC2772561

[46] Calvo S.E., Tucker E.J., Compton A.G., Kirby D.M., Crawford G., Burtt N.P. (2010) High-throughput, pooled sequencing identifies mutations in NUBPL and FOXRED1 in human complex I deficiency. Nat. Genet. 42, 851–858 10.1038/ng.659 20818383 PMC2977978

[47] Andrews B., Carroll J., Ding S., Fearnley I.M. and Walker J.E. (2013) Assembly factors for the membrane arm of human complex I. Proc. Natl. Acad. Sci. U.S.A. 110, 18934–18939 10.1073/pnas.1319247110 24191001 PMC3839705

[48] Guarani V., Paulo J., Zhai B., Huttlin E.L., Gygi S.P. and Harper J.W. (2014) TIMMDC1/C3orf1 functions as a membrane-embedded mitochondrial complex I assembly factor through association with the MCIA complex. Mol. Cell. Biol. 34, 847–861 10.1128/MCB.01551-13 24344204 PMC4023825

[49] Vogel R.O., Janssen R.J., Ugalde C., Grovenstein M., Huijbens R.J., Visch H.J. (2005) Human mitochondrial complex I assembly is mediated by NDUFAF1. FEBS J. 272, 5317–5326 10.1111/j.1742-4658.2005.04928.x 16218961

[50] Dunning C.J., McKenzie M., Sugiana C., Lazarou M., Silke J., Connelly A. (2007) Human CIA30 is involved in the early assembly of mitochondrial complex I and mutations in its gene cause disease. EMBO J. 26, 3227–3237 10.1038/sj.emboj.7601748 17557076 PMC1914096

[51] Vogel R.O., Janssen R.J., van den Brand M.A., Dieteren C.E., Verkaart S., Koopman W.J. (2007) Cytosolic signaling protein Ecsit also localizes to mitochondria where it interacts with chaperone NDUFAF1 and functions in complex I assembly. Genes Dev. 21, 615–624 10.1101/gad.408407 17344420 PMC1820902

[52] Nouws J., Nijtmans L., Houten S.M., van den Brand M., Huynen M., Venselaar H. (2010) Acyl-CoA dehydrogenase 9 is required for the biogenesis of oxidative phosphorylation complex I. Cell Metab. 12, 283–294 10.1016/j.cmet.2010.08.002 20816094

[53] Haack T.B., Danhauser K., Haberberger B., Hoser J., Strecker V., Boehm D. (2010) Exome sequencing identifies ACAD9 mutations as a cause of complex I deficiency. Nat. Genet. 42, 1131–1134 10.1038/ng.706 21057504

[54] Heide H., Bleier L., Steger M., Ackermann J., Drose S., Schwamb B. (2012) Complexome profiling identifies TMEM126B as a component of the mitochondrial complex I assembly complex. Cell Metab. 16, 538–549 10.1016/j.cmet.2012.08.009 22982022

[55] Szklarczyk R., Wanschers B.F., Cuypers T.D., Esseling J.J., Riemersma M., van den Brand M.A. (2012) Iterative orthology prediction uncovers new mitochondrial proteins and identifies C12orf62 as the human ortholog of COX14, a protein involved in the assembly of cytochrome c oxidase. Genome Biol. 13, R12 10.1186/gb-2012-13-2-r12 22356826 PMC3334569

[56] Mick D.U., Dennerlein S., Wiese H., Reinhold R., Pacheu-Grau D., Lorenzi I. (2012) MITRAC links mitochondrial protein translocation to respiratory-chain assembly and translational regulation. Cell 151, 1528–1541 10.1016/j.cell.2012.11.053 23260140

[57] Fassone E., Duncan A.J., Taanman J.W., Pagnamenta A.T., Sadowski M.I., Holand T. (2010) FOXRED1, encoding an FAD-dependent oxidoreductase complex-I-specific molecular chaperone, is mutated in infantile-onset mitochondrial encephalopathy. Hum. Mol. Genet. 19, 4837–4847 10.1093/hmg/ddq414 20858599 PMC4560042

[58] Formosa L.E., Mimaki M., Frazier A.E., McKenzie M., Stait T.L., Thorburn D.R. (2015) Characterization of mitochondrial FOXRED1 in the assembly of respiratory chain complex I. Hum. Mol. Genet. 24, 2952–2965 10.1093/hmg/ddv058 25678554

[59] Zurita Rendon O., Antonicka H., Horvath R. and Shoubridge E.A. (2016) A mutation in the FAD-dependent oxidoreductase FOXRED1 results in cell-type specific assembly defects in oxidative phosphorylation complexes I and II. Mol. Cell. Biol., 10.1128/MCB.00066-16 27215383 PMC4968213

[60] Cizkova A., Stranecky V., Mayr J.A., Tesarova M., Havlickova V., Paul J. (2008) TMEM70 mutations cause isolated ATP synthase deficiency and neonatal mitochondrial encephalocardiomyopathy. Nat. Genet. 40, 1288–1290 10.1038/ng.246 18953340

[61] Hejzlarova K., Mracek T., Vrbacky M., Kaplanova V., Karbanova V., Nuskova H. (2014) Nuclear genetic defects of mitochondrial ATP synthase. Physiol. Res. 63, S57–S71 24564666 10.33549/physiolres.932643

[62] Ogilvie I., Kennaway N.G. and Shoubridge E.A. (2005) A molecular chaperone for mitochondrial complex I assembly is mutated in a progressive encephalopathy. J. Clin. Invest. 115, 2784–2792 10.1172/JCI26020 16200211 PMC1236688

[63] Oyedotun K.S. and Lemire B.D. (2001) The quinone-binding sites of the Saccharomyces cervisiae succinate-ubiquinone oxidoreductase. J. Biol. Chem. 276, 16936–16943 10.1074/jbc.M100184200 11279023

[64] Yankovskaya V., Horsefield R., Tornroth S., Luna-Chavez C., Miyoshi H., Leger C. (2003) Architecture of succinate dehydrogenase and reactive oxygen species generation. Science 299, 700–704 10.1126/science.1079605 12560550

[65] Sun F., Huo X., Zhai Y., Wang A., Xu J., Su D. (2005) Crystal structure of mitochondrial respiratory membrane protein complex II. Cell 121, 1043–1057 10.1016/j.cell.2005.05.025 15989954

[66] Oyedotun K.S., Sit C.S. and Lemire B.D. (2007) The *Saccharomyces cerevisiae* succinate dehydrogenase does not require heme for ubiquinone reduction. Biochim. Biophys. Acta 1767, 1436–1445 10.1016/j.bbabio.2007.09.008 18028869

[67] Hao H.X., Khalimonchuk O., Schraders M., Dephoure N., Bayley J.P., Kunst H. (2009) SDH5, a gene required for flavination of succinate dehydrogenase, is mutated in paraganglioma. Science 325, 1139–1142 10.1126/science.1175689 19628817 PMC3881419

[68] Kim H.J., Jeong M.Y., Na U. and Winge D.R. (2012) Flavinylation and assembly of succinate dehydrogenase are dependent on the C-terminal tail of the flavoprotein subunit. J. Biol. Chem. 287, 40670–40679 10.1074/jbc.M112.405704 23043141 PMC3504780

[69] Van Vranken J.G., Bricker D.K., Dephoure N., Gygi S.P., Cox J.E., Thummel C.S. (2014) SDHAF4 promotes mitochondrial succinate dehydrogenase activity and prevents neurodegeneration. Cell Metab 20, 241–252 10.1016/j.cmet.2014.05.012 24954416 PMC4126880

[70] Braymer J.J. and Lill R. (2017) Iron-sulfur cluster biogenesis and trafficking in mitochondria. J. Biol. Chem. 292, 12754–12763 10.1074/jbc.R117.787101 28615445 PMC5546016

[71] Rouault T.A. and Maio N. (2017) Biogenesis and functions of mammalian iron-sulfur proteins in the regulation of iron homeostasis and pivotal metabolic pathways. J. Biol. Chem. 292, 12744–12753 10.1074/jbc.R117.789537 28615439 PMC5546015

[72] Ghezzi D., Goffrini P., Uziel G., Horvath R., Klopstock T., Lochmuller H. (2009) SDHAF1, encoding a LYR complex-II specific assembly factor, is mutated in SDH-defective infantile leukoencephalopathy. Nat. Genet. 41, 654–656 10.1038/ng.378 19465911

[73] Maio N., Singh A., Uhrigshardt H., Saxena N., Tong W.H. and Rouault T.A. (2014) Cochaperone binding to LYR motifs confers specificity of iron sulfur cluster delivery. Cell Metab. 19, 445–457 10.1016/j.cmet.2014.01.015 24606901 PMC6550293

[74] Maio N., Ghezzi D., Verrigni D., Rizza T., Bertini E., Martinelli D. (2016) Disease-Causing SDHAF1 Mutations Impair Transfer of Fe-S Clusters to SDHB. Cell Metab. 23, 292–302 10.1016/j.cmet.2015.12.005 26749241 PMC4749439

[75] Na U., Yu W., Cox J., Bricker D.K., Brockmann K., Rutter J. (2014) The LYR factors SDHAF1 and SDHAF3 mediate maturation of the iron-sulfur subunit of succinate dehydrogenase. Cell Metab. 20, 253–266 10.1016/j.cmet.2014.05.014 24954417 PMC4126850

[76] Dwight T., Na U., Kim E., Zhu Y., Richardson A.L., Robinson B.G. (2017) Analysis of SDHAF3 in familial and sporadic pheochromocytoma and paraganglioma. BMC Cancer 17, 497 10.1186/s12885-017-3486-z 28738844 PMC5525311

[77] Lemarie A. and Grimm S. (2009) Mutations in the heme b-binding residue of SDHC inhibit assembly of respiratory chain complex II in mammalian cells. Mitochondrion 9, 254–260 10.1016/j.mito.2009.03.004 19332149

[78] Kim H.J., Khalimonchuk O., Smith P.M. and Winge D.R. (2012) Structure, function, and assembly of heme centers in mitochondrial respiratory complexes. Biochim. Biophys. Acta 1823, 1604–1616 10.1016/j.bbamcr.2012.04.008 22554985 PMC3601904

[79] Trumpower B.L. (1990) The protonmotive Q cycle. Energy transduction by coupling of proton translocation to electron transfer by the cytochrome bc1 complex. J. Biol. Chem. 265, 11409–11412 2164001

[80] Crofts A.R., Holland J.T., Victoria D., Kolling D.R., Dikanov S.A., Gilbreth R. (2008) The Q-cycle reviewed: How well does a monomeric mechanism of the bc(1) complex account for the function of a dimeric complex? Biochim. Biophys. Acta 1777, 1001–1019 10.1016/j.bbabio.2008.04.037 18501698 PMC2578832

[81] Iwata S., Lee J.W., Okada K., Lee J.K., Iwata M., Rasmussen B. (1998) Complete structure of the 11-subunit bovine mitochondrial cytochrome bc1 complex. Science 281, 64–71 10.1126/science.281.5373.64 9651245

[82] Hunte C., Koepke J., Lange C., Rossmanith T. and Michel H. (2000) Structure at 2.3 A resolution of the cytochrome bc(1) complex from the yeast Saccharomyces cerevisiae co-crystallized with an antibody Fv fragment. Structure 8, 669–684 10.1016/S0969-2126(00)00152-0 10873857

[83] Brandt U., Yu L., Yu C.A. and Trumpower B.L. (1993) The mitochondrial targeting presequence of the Rieske iron-sulfur protein is processed in a single step after insertion into the cytochrome bc1 complex in mammals and retained as a subunit in the complex. J. Biol. Chem. 268, 8387–8390 8386158

[84] Bottani E., Cerutti R., Harbour M.E., Ravaglia S., Dogan S.A., Giordano C. (2017) TTC19 plays a husbandry role on UQCRFS1 turnover in the biogenesis of mitochondrial respiratory complex III. Mol. Cell. 67, 96.e4–105.e4 10.1016/j.molcel.2017.06.001 28673544

[85] Fernandez-Vizarra E. and Zeviani M. (2018) Mitochondrial complex III Rieske Fe-S protein processing and assembly. Cell Cycle 17, 681–687, 10.1080/15384101.2017. 29243944 PMC5969560

[86] Haut S., Brivet M., Touati G., Rustin P., Lebon S., Garcia-Cazorla A. (2003) A deletion in the human QP-C gene causes a complex III deficiency resulting in hypoglycaemia and lactic acidosis. Hum. Genet. 113, 118–122 12709789 10.1007/s00439-003-0946-0

[87] Barel O., Shorer Z., Flusser H., Ofir R., Narkis G., Finer G. (2008) Mitochondrial complex III deficiency associated with a homozygous mutation in UQCRQ. Am. J. Hum. Genet. 82, 1211–1216 10.1016/j.ajhg.2008.03.020 18439546 PMC2427202

[88] Gruschke S., Kehrein K., Rompler K., Grone K., Israel L., Imhof A. (2011) Cbp3-Cbp6 interacts with the yeast mitochondrial ribosomal tunnel exit and promotes cytochrome b synthesis and assembly. J. Cell Biol. 193, 1101–1114 10.1083/jcb.201103132 21670217 PMC3115798

[89] Gruschke S., Rompler K., Hildenbeutel M., Kehrein K., Kuhl I., Bonnefoy N. (2012) The Cbp3-Cbp6 complex coordinates cytochrome b synthesis with bc(1) complex assembly in yeast mitochondria. J. Cell Biol. 199, 137–150 10.1083/jcb.201206040 23007649 PMC3461508

[90] Hildenbeutel M., Hegg E.L., Stephan K., Gruschke S., Meunier B. and Ott M. (2014) Assembly factors monitor sequential hemylation of cytochrome b to regulate mitochondrial translation. J. Cell Biol. 205, 511–524 10.1083/jcb.201401009 24841564 PMC4033779

[91] Tucker E.J., Wanschers B.F., Szklarczyk R., Mountford H.S., Wijeyeratne X.W., van den Brand M.A. (2013) Mutations in the UQCC1-interacting protein, UQCC2, cause human complex III deficiency associated with perturbed cytochrome b protein expression. PLoS Genet. 9, e1004034 10.1371/journal.pgen.1004034 24385928 PMC3873243

[92] Wanschers B.F., Szklarczyk R., van den Brand M.A., Jonckheere A., Suijskens J., Smeets R. (2014) A mutation in the human CBP4 ortholog UQCC3 impairs complex III assembly, activity and cytochrome b stability. Hum. Mol. Genet. 23, 6356–6365 10.1093/hmg/ddu357 25008109

[93] Cruciat C.M., Hell K., Folsch H., Neupert W. and Stuart R.A. (1999) Bcs1p, an AAA-family member, is a chaperone for the assembly of the cytochrome bc(1) complex. EMBO J. 18, 5226–5233 10.1093/emboj/18.19.5226 10508156 PMC1171593

[94] Fernandez-Vizarra E., Bugiani M., Goffrini P., Carrara F., Farina L., Procopio E. (2007) Impaired complex III assembly associated with BCS1L gene mutations in isolated mitochondrial encephalopathy. Hum. Mol. Genet. 16, 1241–1252 10.1093/hmg/ddm072 17403714

[95] Conte A., Papa B., Ferramosca A. and Zara V. (2015) The dimerization of the yeast cytochrome bc1 complex is an early event and is independent of Rip1. Biochim. Biophys. Acta 1853, 987–995 10.1016/j.bbamcr.2015.02.006 25683140

[96] Atkinson A., Smith P., Fox J.L., Cui T.Z., Khalimonchuk O. and Winge D.R. (2011) The LYR protein Mzm1 functions in the insertion of the Rieske Fe/S protein in yeast mitochondria. Mol. Cell. Biol. 31, 3988–3996 10.1128/MCB.05673-11 21807901 PMC3187353

[97] Cui T.Z., Smith P.M., Fox J.L., Khalimonchuk O. and Winge D.R. (2012) Late-stage maturation of the Rieske Fe/S protein: Mzm1 stabilizes Rip1 but does not facilitate its translocation by the AAA ATPase Bcs1. Mol. Cell. Biol. 32, 4400–4409 10.1128/MCB.00441-12 22927643 PMC3486142

[98] Sanchez E., Lobo T., Fox J.L., Zeviani M., Winge D.R. and Fernandez-Vizarra E. (2013) LYRM7/MZM1L is a UQCRFS1 chaperone involved in the last steps of mitochondrial Complex III assembly in human cells. Biochim. Biophys. Acta 1827, 285–293 10.1016/j.bbabio.2012.11.003 23168492 PMC3570683

[99] Maio N., Kim K.S., Singh A. and Rouault T.A. (2017) A single adaptable Cochaperone-Scaffold complex delivers nascent iron-sulfur clusters to mammalian respiratory chain complexes I-III. Cell Metab. 25, 945e6–953e6 10.1016/j.cmet.2017.03.010 28380382 PMC12285277

[100] de Lonlay P., Valnot I., Barrientos A., Gorbatyuk M., Tzagoloff A., Taanman J.W. (2001) A mutant mitochondrial respiratory chain assembly protein causes complex III deficiency in patients with tubulopathy, encephalopathy and liver failure. Nat. Genet. 29, 57–60 10.1038/ng706 11528392

[101] Wagener N., Ackermann M., Funes S. and Neupert W. (2011) A pathway of protein translocation in mitochondria mediated by the AAA-ATPase Bcs1. Mol. Cell 44, 191–202 10.1016/j.molcel.2011.07.036 22017868

[102] Ghezzi D., Arzuffi P., Zordan M., Da Re C., Lamperti C., Benna C. (2011) Mutations in TTC19 cause mitochondrial complex III deficiency and neurological impairment in humans and flies. Nat. Genet. 43, 259–263 10.1038/ng.761 21278747

[103] Fernandez-Vizarra E. and Zeviani M. (2015) Nuclear gene mutations as the cause of mitochondrial complex III deficiency. Front. Genet. 6, 134 10.3389/fgene.2015.00134 25914718 PMC4391031

[104] Zara V., Palmisano I., Conte L. and Trumpower B.L. (2004) Further insights into the assembly of the yeast cytochrome bc1 complex based on analysis of single and double deletion mutants lacking supernumerary subunits and cytochrome b. Eur. J. Biochem. 271, 1209–1218 10.1111/j.1432-1033.2004.04024.x 15009199

[105] Zara V., Conte L. and Trumpower B.L. (2007) Identification and characterization of cytochrome bc(1) subcomplexes in mitochondria from yeast with single and double deletions of genes encoding cytochrome bc(1) subunits. FEBS J. 274, 4526–4539 10.1111/j.1742-4658.2007.05982.x 17680808

[106] Zara V., Conte L. and Trumpower B.L. (2009) Evidence that the assembly of the yeast cytochrome bc1 complex involves the formation of a large core structure in the inner mitochondrial membrane. FEBS J. 276, 1900–1914 10.1111/j.1742-4658.2009.06916.x 19236481 PMC2745923

[107] Zara V., Conte L. and Trumpower B.L. (2009) Biogenesis of the yeast cytochrome bc1 complex. Biochim. Biophys. Acta 1793, 89–96 10.1016/j.bbamcr.2008.04.011 18501197

[108] Wikstrom M., Krab K. and Sharma V. (2018) Oxygen activation and energy conservation by cytochrome c oxidase. Chem. Rev. 10.1021/acs.chemrev.7b00664 29350917 PMC6203177

[109] Yoshikawa S., Shinzawa-Itoh K. and Tsukihara T. (1998) Crystal structure of bovine heart cytochrome c oxidase at 2.8 A resolution. J. Bioenerg. Biomembr. 30, 7–14 10.1023/A:1020595108560 9623800

[110] Balsa E., Marco R., Perales-Clemente E., Szklarczyk R., Calvo E., Landazuri M.O. (2012) NDUFA4 is a subunit of complex IV of the mammalian electron transport chain. Cell Metab. 16, 378–386 10.1016/j.cmet.2012.07.015 22902835

[111] Kadenbach B. (2017) Regulation of mammalian 13-subunit cytochrome c oxidase and binding of other proteins: role of NDUFA4. Trends Endocrinol. Metab. 28, 761–770 10.1016/j.tem.2017.09.003 28988874

[112] Arnold S., Goglia F. and Kadenbach B. (1998) 3,5-Diiodothyronine binds to subunit Va of cytochrome-c oxidase and abolishes the allosteric inhibition of respiration by ATP. Eur. J. Biochem. 252, 325–330 10.1046/j.1432-1327.1998.2520325.x 9523704

[113] Arnold S. and Kadenbach B. (1997) Cell respiration is controlled by ATP, an allosteric inhibitor of cytochrome-c oxidase. Eur. J. Biochem. 249, 350–354 10.1111/j.1432-1033.1997.t01-1-00350.x 9363790

[114] Kadenbach B. and Arnold S. (1999) A second mechanism of respiratory control. FEBS Lett. 447, 131–134 10.1016/S0014-5793(99)00229-X 10214932

[115] Massa V., Fernandez-Vizarra E., Alshahwan S., Bakhsh E., Goffrini P., Ferrero I. (2008) Severe infantile encephalomyopathy caused by a mutation in COX6B1, a nucleus-encoded subunit of cytochrome c oxidase. Am. J. Hum. Genet. 82, 1281–1289 10.1016/j.ajhg.2008.05.002 18499082 PMC2427282

[116] Fornuskova D., Stiburek L., Wenchich L., Vinsova K., Hansikova H. and Zeman J. (2010) Novel insights into the assembly and function of human nuclear-encoded cytochrome c oxidase subunits 4, 5a, 6a, 7a and 7b. Biochem. J. 428, 363–374 10.1042/BJ20091714 20307258

[117] Pitceathly R.D., Rahman S., Wedatilake Y., Polke J.M., Cirak S., Foley A.R. (2013) NDUFA4 mutations underlie dysfunction of a cytochrome c oxidase subunit linked to human neurological disease. Cell Rep. 3, 1795–1805 10.1016/j.celrep.2013.05.005 23746447 PMC3701321

[118] Huttemann M., Kadenbach B. and Grossman L.I. (2001) Mammalian subunit IV isoforms of cytochrome c oxidase. Gene 267, 111–123 10.1016/S0378-1119(01)00385-7 11311561

[119] Sinkler C.A., Kalpage H., Shay J., Lee I., Malek M.H., Grossman L.I. (2017) Tissue- and condition-specific isoforms of mammalian cytochrome c oxidase subunits: from function to human disease. Oxid. Med. Cell Longev. 2017, 1534056 10.1155/2017/1534056 28593021 PMC5448071

[120] Nijtmans L.G., Taanman J.W., Muijsers A.O., Speijer D. and Van den Bogert C. (1998) Assembly of cytochrome-c oxidase in cultured human cells. Eur. J. Biochem. 254, 389–394 10.1046/j.1432-1327.1998.2540389.x 9660196

[121] Stiburek L., Hansikova H., Tesarova M., Cerna L. and Zeman J. (2006) Biogenesis of eukaryotic cytochrome c oxidase. Physiol. Res. 55 (Suppl. 2), S27–S41 17298220 10.33549/physiolres.930000.55.S2.27

[122] Stiburek L., Vesela K., Hansikova H., Pecina P., Tesarova M., Cerna L. (2005) Tissue-specific cytochrome c oxidase assembly defects due to mutations in SCO2 and SURF1. Biochem. J. 392, 625–632 10.1042/BJ20050807 16083427 PMC1316303

[123] Vidoni S., Harbour M.E., Guerrero-Castillo S., Signes A., Ding S., Fearnley I.M. (2017) MR-1S interacts with PET100 and PET117 in module-based assembly of human cytochrome c oxidase. Cell Rep. 18, 1727–1738 10.1016/j.celrep.2017.01.044 28199844

[124] Timon-Gomez A., Nyvltova E., Abriata L.A., Vila A.J., Hosler J. and Barrientos A. (2018) Mitochondrial cytochrome c oxidase biogenesis: recent developments. Semin. Cell Dev. Biol. 76, 163–178, 10.106/j.semcdb.2017.08.055 28870773 PMC5842095

[125] McStay G.P., Su C.H. and Tzagoloff A. (2013) Modular assembly of yeast cytochrome oxidase. Mol. Biol. Cell 24, 440–452 10.1091/mbc.e12-10-0749 23266989 PMC3571867

[126] Hayashi T., Asano Y., Shintani Y., Aoyama H., Kioka H., Tsukamoto O. (2015) Higd1a is a positive regulator of cytochrome c oxidase. Proc. Natl. Acad. Sci. U.S.A. 112, 1553–1558 10.1073/pnas.1419767112 25605899 PMC4321285

[127] Lundin C., von Ballmoos C., Ott M., Adelroth P. and Brzezinski P. (2016) Regulatory role of the respiratory supercomplex factors in *Saccharomyces cerevisiae*. Proc. Natl. Acad. Sci. U.S.A. 113, E4476–E4485 10.1073/pnas.1601196113 27432958 PMC4978307

[128] Strogolova V., Furness A., Robb-McGrath M., Garlich J. and Stuart R.A. (2012) Rcf1 and Rcf2, members of the hypoxia-induced gene 1 protein family, are critical components of the mitochondrial cytochrome bc1-cytochrome c oxidase supercomplex. Mol. Cell. Biol. 32, 1363–1373 10.1128/MCB.06369-11 22310663 PMC3318584

[129] Vukotic M., Oeljeklaus S., Wiese S., Vogtle F.N., Meisinger C., Meyer H.E. (2012) Rcf1 mediates cytochrome oxidase assembly and respirasome formation, revealing heterogeneity of the enzyme complex. Cell Metab. 15, 336–347 10.1016/j.cmet.2012.01.016 22342701

[130] Dennerlein S., Oeljeklaus S., Jans D., Hellwig C., Bareth B., Jakobs S. (2015) MITRAC7 acts as a COX1-specific chaperone and reveals a checkpoint during cytochrome c oxidase assembly. Cell Rep. 12, 1644–1655 10.1016/j.celrep.2015.08.009 26321642

[131] Weraarpachai W., Sasarman F., Nishimura T., Antonicka H., Aure K., Rotig A. (2012) Mutations in C12orf62, a factor that couples COX I synthesis with cytochrome c oxidase assembly, cause fatal neonatal lactic acidosis. Am. J. Hum. Genet. 90, 142–151 10.1016/j.ajhg.2011.11.027 22243966 PMC3257963

[132] Clemente P., Peralta S., Cruz-Bermudez A., Echevarria L., Fontanesi F., Barrientos A. (2013) hCOA3 stabilizes cytochrome c oxidase 1 (COX1) and promotes cytochrome c oxidase assembly in human mitochondria. J. Biol. Chem. 288, 8321–8331 10.1074/jbc.M112.422220 23362268 PMC3605650

[133] Richter-Dennerlein R., Oeljeklaus S., Lorenzi I., Ronsor C., Bareth B., Schendzielorz A.B. (2016) Mitochondrial protein synthesis adapts to influx of nuclear-encoded protein. Cell 167, 471e10–483e10 10.1016/j.cell.2016.09.003 27693358 PMC5055049

[134] Bourens M. and Barrientos A. (2017) A CMC1-knockout reveals translation-independent control of human mitochondrial complex IV biogenesis. EMBO Rep. 18, 477–494 10.15252/embr.201643103 28082314 PMC5331208

[135] Mootha V.K., Lepage P., Miller K., Bunkenborg J., Reich M., Hjerrild M. (2003) Identification of a gene causing human cytochrome c oxidase deficiency by integrative genomics. Proc. Natl. Acad. Sci. U.S.A. 100, 605–610 10.1073/pnas.242716699 12529507 PMC141043

[136] Xu F., Morin C., Mitchell G., Ackerley C. and Robinson B.H. (2004) The role of the LRPPRC (leucine-rich pentatricopeptide repeat cassette) gene in cytochrome oxidase assembly: mutation causes lowered levels of COX (cytochrome c oxidase) I and COX III mRNA. Biochem. J. 382, 331–336 10.1042/BJ20040469 15139850 PMC1133946

[137] Ruzzenente B., Metodiev M.D., Wredenberg A., Bratic A., Park C.B., Camara Y. (2012) LRPPRC is necessary for polyadenylation and coordination of translation of mitochondrial mRNAs. EMBO J. 31, 443–456 10.1038/emboj.2011.392 22045337 PMC3261557

[138] Weraarpachai W., Antonicka H., Sasarman F., Seeger J., Schrank B., Kolesar J.E. (2009) Mutation in TACO1, encoding a translational activator of COX I, results in cytochrome c oxidase deficiency and late-onset Leigh syndrome. Nat. Genet. 41, 833–837 10.1038/ng.390 19503089

[139] Richman T.R., Spahr H., Ermer J.A., Davies S.M., Viola H.M., Bates K.A. (2016) Loss of the RNA-binding protein TACO1 causes late-onset mitochondrial dysfunction in mice. Nat. Commun. 7, 11884 10.1038/ncomms11884 27319982 PMC4915168

[140] Antonicka H., Leary S.C., Guercin G.H., Agar J.N., Horvath R., Kennaway N.G. (2003) Mutations in COX10 result in a defect in mitochondrial heme A biosynthesis and account for multiple, early-onset clinical phenotypes associated with isolated COX deficiency. Hum. Mol. Genet. 12, 2693–2702 10.1093/hmg/ddg284 12928484

[141] Diaz F., Thomas C.K., Garcia S., Hernandez D. and Moraes C.T. (2005) Mice lacking COX10 in skeletal muscle recapitulate the phenotype of progressive mitochondrial myopathies associated with cytochrome c oxidase deficiency. Hum. Mol. Genet. 14, 2737–2748 10.1093/hmg/ddi307 16103131 PMC2778476

[142] Antonicka H., Mattman A., Carlson C.G., Glerum D.M., Hoffbuhr K.C., Leary S.C. (2003) Mutations in COX15 produce a defect in the mitochondrial heme biosynthetic pathway, causing early-onset fatal hypertrophic cardiomyopathy. Am. J. Hum. Genet. 72, 101–114 10.1086/345489 12474143 PMC378614

[143] Tiranti V., Hoertnagel K., Carrozzo R., Galimberti C., Munaro M., Granatiero M. (1998) Mutations of SURF-1 in Leigh disease associated with cytochrome c oxidase deficiency. Am. J. Hum. Genet. 63, 1609–1621 10.1086/302150 9837813 PMC1377632

[144] Zhu Z., Yao J., Johns T., Fu K., De Bie I., Macmillan C. (1998) SURF1, encoding a factor involved in the biogenesis of cytochrome c oxidase, is mutated in Leigh syndrome. Nat. Genet. 20, 337–343 10.1038/3804 9843204

[145] Taylor N.G., Swenson S., Harris N.J., Germany E.M., Fox J.L. and Khalimonchuk O. (2017) The assembly factor Pet117 couples heme a synthase activity to cytochrome oxidase assembly. J. Biol. Chem. 292, 1815–1825 10.1074/jbc.M116.766980 27998984 PMC5290955

[146] Hiser L., Di Valentin M., Hamer A.G. and Hosler J.P. (2000) Cox11p is required for stable formation of the Cu(B) and magnesium centers of cytochrome c oxidase. J. Biol. Chem. 275, 619–623 10.1074/jbc.275.1.619 10617659

[147] Banci L., Bertini I., Cantini F., Ciofi-Baffoni S., Gonnelli L. and Mangani S. (2004) Solution structure of Cox11, a novel type of beta-immunoglobulin-like fold involved in CuB site formation of cytochrome c oxidase. J. Biol. Chem. 279, 34833–34839 10.1074/jbc.M403655200 15181013

[148] Cobine P.A., Pierrel F. and Winge D.R. (2006) Copper trafficking to the mitochondrion and assembly of copper metalloenzymes. Biochim. Biophys. Acta 1763, 759–772 10.1016/j.bbamcr.2006.03.002 16631971

[149] Glerum D.M., Shtanko A. and Tzagoloff A. (1996) Characterization of COX17, a yeast gene involved in copper metabolism and assembly of cytochrome oxidase. J. Biol. Chem. 271, 14504–14509 10.1074/jbc.271.24.14504 8662933

[150] Bode M., Woellhaf M.W., Bohnert M., van der Laan M., Sommer F., Jung M. (2015) Redox-regulated dynamic interplay between Cox19 and the copper-binding protein Cox11 in the intermembrane space of mitochondria facilitates biogenesis of cytochrome c oxidase. Mol. Biol. Cell 26, 2385–2401 10.1091/mbc.e14-11-1526 25926683 PMC4571295

[151] Pierrel F., Bestwick M.L., Cobine P.A., Khalimonchuk O., Cricco J.A. and Winge D.R. (2007) Coa1 links the Mss51 post-translational function to Cox1 cofactor insertion in cytochrome c oxidase assembly. EMBO J. 26, 4335–4346 10.1038/sj.emboj.7601861 17882260 PMC2034670

[152] Bourens M. and Barrientos A. (2017) Human mitochondrial cytochrome c oxidase assembly factor COX18 acts transiently as a membrane insertase within the subunit 2 maturation module. J. Biol. Chem. 292, 7774–7783 10.1074/jbc.M117.778514 28330871 PMC5427259

[153] Szklarczyk R., Wanschers B.F., Nijtmans L.G., Rodenburg R.J., Zschocke J., Dikow N. (2013) A mutation in the FAM36A gene, the human ortholog of COX20, impairs cytochrome c oxidase assembly and is associated with ataxia and muscle hypotonia. Hum. Mol. Genet. 22, 656–667 10.1093/hmg/dds473 23125284

[154] Bourens M., Boulet A., Leary S.C. and Barrientos A. (2014) Human COX20 cooperates with SCO1 and SCO2 to mature COX2 and promote the assembly of cytochrome c oxidase. Hum. Mol. Genet. 23, 2901–2913 10.1093/hmg/ddu003 24403053 PMC4014192

[155] Lorenzi I., Oeljeklaus S., Aich A., Ronsor C., Callegari S., Dudek J. (2018) The mitochondrial TMEM177 associates with COX20 during COX2 biogenesis. Biochim. Biophys. Acta 1865, 323–333 10.1016/j.bbamcr.2017.11.010 29154948 PMC5764226

[156] Leary S.C., Kaufman B.A., Pellecchia G., Guercin G.H., Mattman A., Jaksch M. (2004) Human SCO1 and SCO2 have independent, cooperative functions in copper delivery to cytochrome c oxidase. Hum. Mol. Genet. 13, 1839–1848 10.1093/hmg/ddh197 15229189

[157] Leary S.C., Cobine P.A., Kaufman B.A., Guercin G.H., Mattman A., Palaty J. (2007) The human cytochrome c oxidase assembly factors SCO1 and SCO2 have regulatory roles in the maintenance of cellular copper homeostasis. Cell Metab. 5, 9–20 10.1016/j.cmet.2006.12.001 17189203

[158] Leary S.C., Sasarman F., Nishimura T. and Shoubridge E.A. (2009) Human SCO2 is required for the synthesis of CO II and as a thiol-disulphide oxidoreductase for SCO1. Hum. Mol. Genet. 18, 2230–2240 10.1093/hmg/ddp158 19336478

[159] Pacheu-Grau D., Bareth B., Dudek J., Juris L., Vogtle F.N., Wissel M. (2015) Cooperation between COA6 and SCO2 in COX2 maturation during cytochrome c oxidase assembly links two mitochondrial cardiomyopathies. Cell Metab. 21, 823–833 10.1016/j.cmet.2015.04.012 25959673

[160] Stroud D.A., Maher M.J., Lindau C., Vogtle F.N., Frazier A.E., Surgenor E. (2015) COA6 is a mitochondrial complex IV assembly factor critical for biogenesis of mtDNA-encoded COX2. Hum. Mol. Genet. 24, 5404–5415 10.1093/hmg/ddv265 26160915

[161] Ghosh A., Pratt A.T., Soma S., Theriault S.G., Griffin A.T., Trivedi P.P. (2016) Mitochondrial disease genes COA6, COX6B and SCO2 have overlapping roles in COX2 biogenesis. Hum. Mol. Genet. 25, 660–671 10.1093/hmg/ddv503 26669719 PMC4743686

[162] Carlson C.G., Barrientos A., Tzagoloff A. and Glerum D.M. (2003) COX16 encodes a novel protein required for the assembly of cytochrome oxidase in Saccharomyces cerevisiae. J. Biol. Chem. 278, 3770–3775 10.1074/jbc.M209893200 12446688

[163] Aich A., Wang C., Chowdhury A., Ronsor C., Pacheu-Grau D., Richter-Dennerlein R. (2018) COX16 promotes COX2 metallation and assembly during respiratory complex IV biogenesis. Elife 7, 10.7554/eLife.32572 29381136 PMC5809144

[164] Cerqua C., Morbidoni V., Desbats M.A., Doimo M., Frasson C., Sacconi S. (2018) COX16 is required for assembly of cytochrome c oxidase in human cells and is involved in copper delivery to COX2. Biochim. Biophys. Acta 1859, 244–252 10.1016/j.bbabio.2018.01.004 29355485

[165] Church C., Goehring B., Forsha D., Wazny P. and Poyton R.O. (2005) A role for Pet100p in the assembly of yeast cytochrome c oxidase: interaction with a subassembly that accumulates in a pet100 mutant. J. Biol. Chem. 280, 1854–1863 10.1074/jbc.M410726200 15507444

[166] Lim S.C., Smith K.R., Stroud D.A., Compton A.G., Tucker E.J., Dasvarma A. (2014) A founder mutation in PET100 causes isolated complex IV deficiency in Lebanese individuals with Leigh syndrome. Am. J. Hum. Genet. 94, 209–222 10.1016/j.ajhg.2013.12.015 24462369 PMC3928654

[167] Olahova M., Haack T.B., Alston C.L., Houghton J.A., He L., Morris A.A. (2015) A truncating PET100 variant causing fatal infantile lactic acidosis and isolated cytochrome c oxidase deficiency. Eur. J. Hum. Genet. 23, 935–939 10.1038/ejhg.2014.214 25293719 PMC4305338

[168] McEwen J.E., Hong K.H., Park S. and Preciado G.T. (1993) Sequence and chromosomal localization of two PET genes required for cytochrome c oxidase assembly in *Saccharomyces cerevisiae*. Curr. Genet. 23, 9–14 10.1007/BF00336742 8381337

[169] Renkema G.H., Visser G., Baertling F., Wintjes L.T., Wolters V.M., van Montfrans J. (2017) Mutated PET117 causes complex IV deficiency and is associated with neurodevelopmental regression and medulla oblongata lesions. Hum. Genet. 136, 759–769 10.1007/s00439-017-1794-7 28386624 PMC5429353

[170] Carroll J., Fearnley I.M., Skehel J.M., Shannon R.J., Hirst J. and Walker J.E. (2006) Bovine complex I is a complex of 45 different subunits. J. Biol. Chem. 281, 32724–32727 10.1074/jbc.M607135200 16950771

[171] Walker J.E. (2013) The ATP synthase: the understood, the uncertain and the unknown. Biochem. Soc. Trans. 41, 1–16 10.1042/BST20110773 23356252

[172] Jonckheere A.I., Smeitink J.A. and Rodenburg R.J. (2012) Mitochondrial ATP synthase: architecture, function and pathology. J. Inherit. Metab. Dis. 35, 211–225 10.1007/s10545-011-9382-9 21874297 PMC3278611

[173] Watt I.N., Montgomery M.G., Runswick M.J., Leslie A.G. and Walker J.E. (2010) Bioenergetic cost of making an adenosine triphosphate molecule in animal mitochondria. Proc. Natl. Acad. Sci. U.S.A. 107, 16823–16827 10.1073/pnas.1011099107 20847295 PMC2947889

[174] Nijtmans L.G., Klement P., Houstek J. and Van den Bogert C. (1995) Assembly of mitochondrial ATP synthase in cultured human cells: implications for mitochondrial diseases. Biochim. Biophys. Acta 1272, 190–198 10.1016/0925-4439(95)00087-9 8541352

[175] Carrozzo R., Wittig I., Santorelli F.M., Bertini E., Hofmann S., Brandt U. (2006) Subcomplexes of human ATP synthase mark mitochondrial biosynthesis disorders. Ann. Neurol. 59, 265–275 10.1002/ana.20729 16365880

[176] Wittig I., Meyer B., Heide H., Steger M., Bleier L., Wumaier Z. (2010) Assembly and oligomerization of human ATP synthase lacking mitochondrial subunits a and A6L. Biochim. Biophys. Acta 1797, 1004–1011 10.1016/j.bbabio.2010.02.021 20188060

[177] Fujikawa M., Sugawara K., Tanabe T. and Yoshida M. (2015) Assembly of human mitochondrial ATP synthase through two separate intermediates, F1-c-ring and b-e-g complex. FEBS Lett. 589, 2707–2712 10.1016/j.febslet.2015.08.006 26297831

[178] He J., Carroll J., Ding S., Fearnley I.M. and Walker J.E. (2017) Permeability transition in human mitochondria persists in the absence of peripheral stalk subunits of ATP synthase. Proc. Natl. Acad. Sci. U.S.A. 114, 9086–9091 10.1073/pnas.1711201114 28784775 PMC5576841

[179] He J., Ford H.C., Carroll J., Ding S., Fearnley I.M. and Walker J.E. (2017) Persistence of the mitochondrial permeability transition in the absence of subunit c of human ATP synthase. Proc. Natl. Acad. Sci. U.S.A. 114, 3409–3414 10.1073/pnas.1702357114 28289229 PMC5380099

[180] He J., Ford H.C., Carroll J., Douglas C., Gonzales E., Ding S. (2018) Assembly of the membrane domain of ATP synthase in human mitochondria. Proc. Natl. Acad. Sci. U.S.A. 10.1073/pnas.1722086115 PMC5866602 29440398

[181] Ackerman S.H. and Tzagoloff A. (1990) Identification of two nuclear genes (ATP11, ATP12) required for assembly of the yeast F1-ATPase. Proc. Natl Acad. Sci. U.S.A. 87, 4986–4990 10.1073/pnas.87.13.4986 2142305 PMC54246

[182] Wang Z.G. and Ackerman S.H. (2000) The assembly factor Atp11p binds to the beta-subunit of the mitochondrial F(1)-ATPase. J. Biol. Chem. 275, 5767–5772 10.1074/jbc.275.8.5767 10681564

[183] Wang Z.G., Sheluho D., Gatti D.L. and Ackerman S.H. (2000) The alpha-subunit of the mitochondrial F(1) ATPase interacts directly with the assembly factor Atp12p. EMBO J. 19, 1486–1493 10.1093/emboj/19.7.1486 10747017 PMC310218

[184] Wang Z.G., White P.S. and Ackerman S.H. (2001) Atp11p and Atp12p are assembly factors for the F(1)-ATPase in human mitochondria. J. Biol. Chem. 276, 30773–30778 10.1074/jbc.M104133200 11410595

[185] De Meirleir L., Seneca S., Lissens W., De Clercq I., Eyskens F., Gerlo E. (2004) Respiratory chain complex V deficiency due to a mutation in the assembly gene ATP12. J. Med. Genet. 41, 120–124 10.1136/jmg.2003.012047 14757859 PMC1735674

[186] Magner M., Dvorakova V., Tesarova M., Mazurova S., Hansikova H., Zahorec M. (2015) TMEM70 deficiency: long-term outcome of 48 patients. J. Inherit. Metab. Dis. 38, 417–426 10.1007/s10545-014-9774-8 25326274

[187] Wittig I. and Schagger H. (2008) Structural organization of mitochondrial ATP synthase. Biochim. Biophys. Acta 1777, 592–598 10.1016/j.bbabio.2008.04.027 18485888

[188] Mourier A., Matic S., Ruzzenente B., Larsson N.G. and Milenkovic D. (2014) The respiratory chain supercomplex organization is independent of COX7a2l isoforms. Cell Metab. 20, 1069–1075 10.1016/j.cmet.2014.11.005 25470551 PMC4261080

[189] Guo R., Zong S., Wu M., Gu J. and Yang M. (2017) Architecture of human mitochondrial respiratory megacomplex I2III2IV2. Cell 170, 1247.e12–1257.e12 10.1016/j.cell.2017.07.050 28844695

[190] Gu J., Wu M., Guo R., Yan K., Lei J., Gao N. (2016) The architecture of the mammalian respirasome. Nature 537, 639–643 10.1038/nature19359 27654917

[191] Wu M., Gu J., Guo R., Huang Y. and Yang M. (2016) Structure of mammalian respiratory supercomplex I1III2IV1. Cell 167, 1598.e10–609.e10 10.1016/j.cell.2016.11.012 27912063

[192] Letts J.A., Fiedorczuk K. and Sazanov L.A. (2016) The architecture of respiratory supercomplexes. Nature 537, 644–648 10.1038/nature19774 27654913

[193] Sousa J.S., Mills D.J., Vonck J. and Kuhlbrandt W. (2016) Functional asymmetry and electron flow in the bovine respirasome. Elife 5, 10.7554/eLife.21290 27830641 PMC5117854

[194] Lapuente-Brun E., Moreno-Loshuertos R., Acin-Perez R., Latorre-Pellicer A., Colas C., Balsa E. (2013) Supercomplex assembly determines electron flux in the mitochondrial electron transport chain. Science 340, 1567–1570 10.1126/science.1230381 23812712

[195] Moreno-Lastres D., Fontanesi F., Garcia-Consuegra I., Martin M.A., Arenas J., Barrientos A. (2012) Mitochondrial complex I plays an essential role in human respirasome assembly. Cell Metab. 15, 324–335 10.1016/j.cmet.2012.01.015 22342700 PMC3318979

[196] Williams E.G., Wu Y., Jha P., Dubuis S., Blattmann P., Argmann C.A. (2016) Systems proteomics of liver mitochondria function. Science 352, aad0189 10.1126/science.aad0189 27284200 PMC10859670

[197] Perez-Perez R., Lobo-Jarne T., Milenkovic D., Mourier A., Bratic A., Garcia-Bartolome A. (2016) COX7A2L is a mitochondrial complex III binding protein that stabilizes the III2+IV supercomplex without affecting respirasome formation. Cell Rep. 16, 2387–2398 10.1016/j.celrep.2016.07.081 27545886 PMC5007171

[198] Cogliati S., Calvo E., Loureiro M., Guaras A.M., Nieto-Arellano R., Garcia-Poyatos C. (2016) Mechanism of super-assembly of respiratory complexes III and IV. Nature 539, 579–582 10.1038/nature20157 27775717

[199] Formosa L.E., Dibley M.G., Stroud D.A. and Ryan M.T. (2018) Building a complex complex: assembly of mitochondrial respiratory chain complex I. Semin. Cell Dev. Biol., 76, 154–162 28797839 10.1016/j.semcdb.2017.08.011

[200] Zhou A., Rohou A., Schep D.G., Bason J.V., Montgomery M.G., Walker J.E. (2015) Structure and conformational states of the bovine mitochondrial ATP synthase by cryo-EM. Elife 4, e10180 10.7554/eLife.10180 26439008 PMC4718723

[201] Ghezzi D., Zeviani M. (2018) Human diseases associated with defects in assembly of OXPHOS complexes. Essays Biochem. 62, 271–286 10.1042/EBC20170099 30030362 PMC6056716

